# Multi-layer perceptron classification & quantification of neuronal survival in hypoxic-ischemic brain image slices using a novel gradient direction, grey level co-occurrence matrix image training

**DOI:** 10.1371/journal.pone.0278874

**Published:** 2022-12-13

**Authors:** Saheli Bhattacharya, Laura Bennet, Joanne O. Davidson, Charles P. Unsworth

**Affiliations:** 1 Department of Engineering Science, The University of Auckland, Auckland, New Zealand; 2 Department of Physiology, The University of Auckland, Auckland, New Zealand; Taipei Medical University, TAIWAN

## Abstract

Hypoxic ischemic encephalopathy (HIE) is a major global cause of neonatal death and lifelong disability. Large animal translational studies of hypoxic ischemic brain injury, such as those conducted in fetal sheep, have and continue to play a key role in furthering our understanding of the cellular and molecular mechanisms of injury and developing new treatment strategies for clinical translation. At present, the quantification of neurons in histological images consists of slow, manually intensive morphological assessment, requiring many repeats by an expert, which can prove to be time-consuming and prone to human error. Hence, there is an urgent need to automate the neuron classification and quantification process. In this article, we present a ’Gradient Direction, Grey level Co-occurrence Matrix’ (GD-GLCM) image training method which outperforms and simplifies the standard training methodology using texture analysis to cell-classification. This is achieved by determining the Grey level Co-occurrence Matrix of the gradient direction of a cell image followed by direct passing to a classifier in the form of a Multilayer Perceptron (MLP). Hence, avoiding all texture feature computation steps. The proposed MLP is trained on both healthy and dying neurons that are manually identified by an expert and validated on unseen hypoxic-ischemic brain slice images from the fetal sheep *in utero* model. We compared the performance of our classifier using the gradient magnitude dataset as well as the gradient direction dataset. We also compare the performance of a perceptron, a 1-layer MLP, and a 2-layer MLP to each other. We demonstrate here a way of accurately identifying both healthy and dying cortical neurons obtained from brain slice images of the fetal sheep model under global hypoxia to high precision by identifying the most minimised MLP architecture, minimised input space (GLCM size) and minimised training data (GLCM representations) to achieve the highest performance over the standard methodology.

## 1. Introduction

Loss of oxygen (hypoxia) and blood (ischemia) supply to the brain either before, during or shortly after labour can result in death or brain damage, termed hypoxic ischemic encephalopathy (HIE). There are a variety of factors that can cause perinatal hypoxia ischemia, including maternal haemorrhage or hypotension, placental insufficiency, a knot in the umbilical cord, prolonged labour or neonatal cardiovascular collapse [1]. Moderate to severe HIE occurs in approximately 2/1000 live term births, with approximately 15–60% of affected neonates dying and 25% of survivors having a long-term disability [2]. These disabilities may include cerebral palsy [3–5], visual impairment and hearing loss, cognitive delay [6], language disorders, microcephaly, and muscle spasticity [7, 8] and epilepsy [6]. Despite high morbidity rates among the HIE survivors, available treatments remain quite limited [9, 10]. The only available treatment to significantly reduce death and disability after moderate to severe HIE is therapeutic hypothermia (brain cooling) [11]. The benefits of therapeutic hypothermia on reducing death and disability have been shown to persist into mid-childhood [12]. However, despite treatment with therapeutic hypothermia, many infants will still develop substantial disabilities [11]. Therefore, developing additional treatment strategies to further reduce this burden of disability is crucial.

The use of large animal translational models and the assessment of histopathological injury was critical to the development of therapeutic hypothermia and will also be critical to the development of novel treatment strategies [13]. Many preclinical studies have been undertaken investigating promising neuroprotective treatments using chronically instrumented fetal sheep preparations, for example investigating the effect of connexin hemichannel blockade [14], recombinant erythropoietin [15] and creatine supplementation [16]. In addition to assessing the recovery of electrophysiological parameters, such as the electroencephalogram, these studies are heavily reliant on the assessment of the survival of key cell types, such as cortical and subcortical neurons, to determine the extent of brain injury and whether or not the treatment of interest was neuroprotective.

In recent years, the application of Machine Learning (ML) and neural inspired ML approaches, known as ’Artificial Neural Networks’ (ANN), has accelerated in the medical and biology fields aiding in the diagnosis and analysis of a wide range of diseases and medical phenomena from different type of medical images [17, 18]. Researchers have employed ML to detect and classify structural brain disorders from images over the last decade [19]. ML has been adapted to detect and classify various brain diseases like Alzheimer’s disease, mild cognitive impairment, Parkinson’s disease, epilepsy, traumatic brain injury and stroke from images [20–23]. Further, ML methods have been successfully adapted to cellular level image data to classify brain tumours [24], detect the severity of cerebral small vessel disease [25], study post-stroke neural connectivity and neuroinflammation [26] and identify ischemic stroke features [27]. The CNN based ANN methods, trained with cellular morphology, have been implemented to identify glia cells [29] and neurons in *ex-vivo* brain images [30]. A Multi-layer-perceptron (MLP) has been employed to classify brain cells exposed to EMF radiation in the model organism Drosophila Melaganaster [28].

Texture analysis (TA) provides information on the pixel inter-relationships and spatial patterns within an image that might be indiscernible to the human eye [29]. With regards to the brain, TA has been used successfully on brain-MRI images to extract features [29, 30] and to detect and classify brain tumours [31–34], breast carcinoma [35], detect ischemic stroke lesions from CT images [36]. The gradient magnitude and direction have been used in the segmentation [37, 38], classification [39, 40], image recognition [41] and pattern detection and classification [42, 43] in an image. The grey-level co-occurrence matrix (GLCM) is a second-order statistical texture analysis method to describe an image’s local heterogeneity information [44–46]. As the definition of GLCM, it characterises the texture by computing how often a pair of pixels of specific values, specified in a given spatial relation (at a given distance and direction), occur in an image [45, 47]. Studies have been conducted on combining GLCM and CNN for various medical diagnosis purposes [48, 49] and forensic research [50, 51]. Researchers have also employed methods using local features like GLCM and LBP (local binary pattern) and a combination of these with CNN [52] to analyse histopathological images. However, the automated classification of HIE cells in histological images has not been eventuated.

The motivation for this work is as follows. Our group is involved in studying the effects of HI (Hypoxia Ischemia) in the term equivalent fetal sheep model [13, 53–55]. Assessing neuronal survival is a vital component of these studies [56, 57]. Some of our recent studies show that the severity of the insult can be correlated with neuronal survival seven days after the HI insult in near term fetal sheep [57, 58]. During the histology assessment of injury in the fetal sheep model, the number of healthy cells (neurons) present in a histological image of a brain slice is typically counted manually and based on the morphological assessment to determine the extent of brain damage in each animal. There are several drawbacks to manual assessment. These are the inevitable variability in cell classification, as human perception is subjective, varying from one evaluator to another; thus, the measurements are subject to intra and inter-rater variability [59, 60]. The manual counting process is also a time-consuming and laborious process requiring multiple repeats, typically taking weeks. Hence, the main motivation for this work is to produce an automated hypoxic-ischemic cell classification and quantification prediction method to rapidly improve speed and accuracy for HI histological images from the *in utero* sheep model by training on known priori’s about the morphology of healthy and dying neuronal cells.

We achieve this by training an Artificial Neural Network (ANN) in the form of a Multilayer perceptron (MLP) on the images of healthy and dying cells that have been identified manually by a human expert. Additional obstacles that we face in the image preprocessing stage before passing to the classifier are as follows. The first obstacle is that the image colour values can depend on the staining procedure rather than the cells’ characteristics themselves. Therefore, it will be better not to use image colour values directly as the input space to our classifier. As the gradient tends to be less influenced by global changes in staining intensity we choose to compute the gradient vectors (gradient-magnitude and gradient direction) of each brain cell and use the gradient maps for further processing.

The second obstacle is that the input space for an MLP requires that all input features be of the same fixed size. However, the cell size and shape is not constant and can vary significantly throughout the images. We will address this in a novel way by transforming each pixelated image to provide uniform input features of the same size by computing the grey-level co-occurrence matrix (GLCM) from each cell’s gradient-magnitude and gradient-direction maps. So, by exploiting the nature of the GLCM, namely that GLCM size is dependent on the grey-scale levels that are user-defined and independent of the size of an image. Therefore, providing the MLP with the same fixed user-defined input space for any size cell.

Thus, in this article, we train an MLP network to classify healthy from dying cells and use two texture analysis approaches (computing gradient magnitude/direction and GLCM) to preprocess the image for effective training. Our prime objective of all preprocessing and ANN design is to determine the most minimised architecture and minimised input data that provides the highest performance.

## 2. Materials and methods

**Fig 1:**
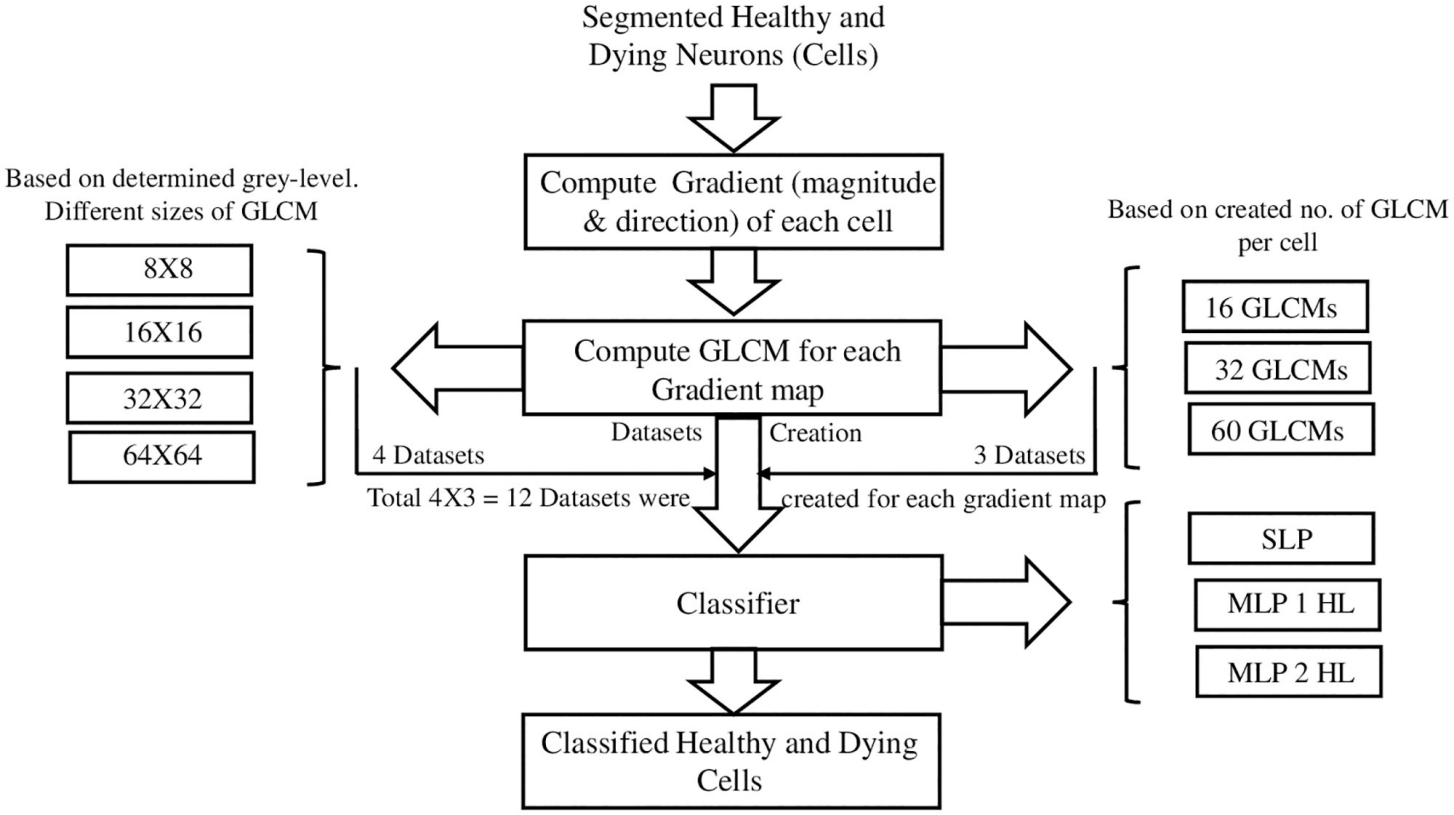
Schematic of the proposed GD-GLCM image training method. SLP denotes Single-Layer Perceptron, MLP denotes Multi-Layers Perceptron, and HL denotes Hidden Layers.

This section will describe the data acquisition process and the proposed methodology to achieve the classification between healthy and dying brain cells of a fetal sheep exposed to global hypoxia *in utereo*. RGB images were taken from the cortex region of a stained sheep brain. Texture analysis was applied as image preprocessing, and SLP (Single layer perceptron) and MLP networks were used as the classifier. A schematic of the proposed methodology for image preprocessing and classification is shown below (Fig 1).

### 2.1. Data acquisition

All procedures were approved by The Animal Ethics Committee of The University of Auckland under the New Zealand Animal Welfare Act and the Code of Ethical Conduct for animals in research, established by the Ministry of Primary Industries, Government of New Zealand. Surgical, experimental and post-mortem procedures were conducted as previously published [14, 61]. The ethics approval number is AEC 22069. In brief, anaesthetised time-mated Romney/Suffolk fetal sheep (n = 17) were instrumented with a variety of catheters and electrodes using sterile techniques at 118 to 124 days of gestation (the term is 145 [57]). The vertebral occipital anastamoses were ligated, and silicon carotid artery occluders were placed loosely around both carotid arteries. After instrumentation, the fetus and ewe were given 4–5 days to recover from anaesthesia and were housed in a metabolic crate. At 128 ±1 day of gestation, global cerebral ischemia was induced by reversible inflation of the carotid occluder cuffs with sterile saline for 30 minutes. Successful occlusion was confirmed by the onset of an isoelectric EEG signal within 30 seconds of inflation [56, 57]. Fetuses and ewes were killed 7 days after hypoxia ischemia by an overdose of sodium pentobarbital (300 mg/mL Pentobarb 300; Provet NZ Pty., Auckland, New Zealand).

#### 2.1.1. Immunohistochemistry

**Fig 2:**
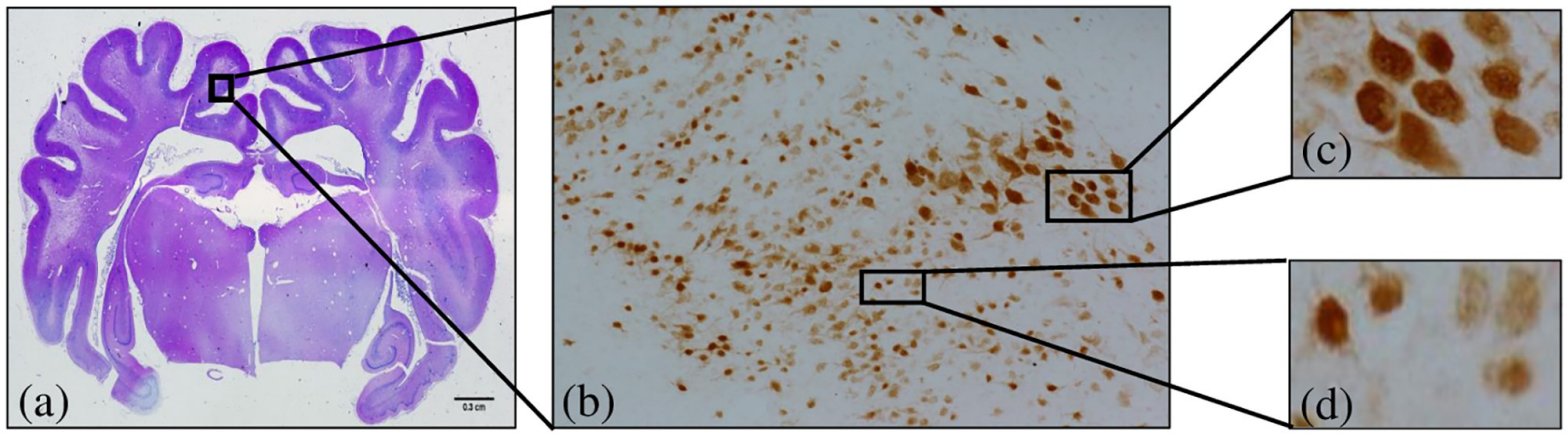
Cortical brain region image of a fetal term sheep exposed to global hypoxia ischemia (a) shows sampling region in the parasagittal cortex of the near-term fetal sheep brain, (b) shows a NeuN- positive stained image of the cortical brain region of a term sheep exposed to global hypoxia consisting both healthy and dying cells, (c) images of healthy cells and, (d) images of dying cells.

Microscopic images (Fig 2) were taken to assess the neuronal survival in the animals exposed to global ischemia 7 days after global cerebral ischemia.

The immunohistochemistry protocol was as follows:

- Fix fetal brains in 10% phosphate-buffered formalin.
- Cut 10 μm thick coronal slices with microtome (Leica Jung RM2035, Wetzler, Germany) starting at the level of the dorsal hippocampus.
- Dewax slides in xylene and rehydrate in decreasing concentrations of ethanol. Wash in 0.1 mol/L phosphate-buffered saline (PBS) for neuronal nuclear antigen (NeuN).
- Perform antigen retrieval using the pressure cooker method (2100 Antigen Retriever, Aptum, Southampton, England) in citrate buff (450 ml dist. H2O, 8 mL citric acid, 42 mL sodium citrate).
- Block endogenous peroxidase activity by incubating in 1% H2O in methanol for 30 mins. (Blocking performed by 3% normal goat serum (NGS) for 1 hour at room temp).
- Label sections with 1:200 rabbit anti-NeuN monoclonal antibody (NeuN, Abcam, Cambridge, England) overnight at 4°C.
- Incubate sections for 3 hours in biotin-conjugated 1:200 inanti-rabbit lgG antibody (Vector Laboratories, Burlingame, USA) in 3% NGS.
- Incubate slides in Extravidin^®^ (1:200) in PBS for 2 hrs at room temp and then react in diaminobenzidine tetrachloride (Sigma-Aldrich Pty. Ltd). Stop reaction by washing in PBS.
- Dehydrate sections in increasing concentrations of alcohol and mount [56, 57]. The neurons are stained red with a slightly darker red nucleus.

Fig 2 shows an image of the cortical brain region of a fetal term sheep exposed to global ischemia consisting both the healthy and dying neurons/cells.

Our data set is created of cells segmented from a total of 68 images of 17 sheep. The cells from the original images are identified and marked by an expert in the field. The cells are then segmented automatically using the method described in our earlier paper [62]. A total of 500 healthy cells and 500 dying cells segmented from 68 brain slice images were used for our classification study. Measurements were taken to make sure that no other cells or parts of other cells were included in the segmented cell images. The datasets generated by our group are highly specialised and require chronically instrumented *in utero* sheep models to generate such hypoxic ischemic brain slices for images to be taken. Thus, data sets are not available publicly.

### 2.2. Texture analysis pre-processing

#### 2.2.1. Gradient- magnitude (GM) & gradient-direction (GD)

If the gradient or first derivative of a 2D function *f*(*x*,*y*) is (*g*_*xy*_) then the gradient vector, its magnitude (mag(*g*_*xy*_)) and direction *α*(*x*,*y*) can be defined in equation [41, 42] respectively:

$$g_{xy}=\;\left[\begin{array}{c} g_{x}\\ g_{y} \end{array}\right]=\;\left[\begin{array}{c} \frac{\partial f}{\partial x}\\ \frac{\partial f}{\partial y} \end{array}\right]$$ (1)

maggxy=gx2+gy212 (2)

$$\alpha \left(x,y\right)=\;{\text{tan}}^{-1}\left[\frac{g_{x}}{g_{y}}\right]$$ (3)

As the gradient is less prone to be influenced by global changes in staining intensity, we computed the gradient vector of each neuron than directly using the image colour value. The colour-coded map of GM was obtained to identify the pattern difference between healthy and dying cells. In addition, the GD was also determined for comparison. For the purpose of our research, the gradient-magnitude and gradient-direction values were rescaled to (N) grey levels (where N = 8, 16, 32 and 64) and were used to compute the GLCMs.

#### 2.2.2. Grey-level co-occurrence matrix (GLCM)

A grey-level co-occurrence matrix (GLCM) is a statistical method used to analyse the texture of an image. It characterises the texture by computing how often a pair of pixels of specific values specified in a given spatial relation (at a given distance and direction) occur in an image [47, 63]. If an image *f*(*x*,*y*) has (N) grey levels, the co-occurrence matrix value *p*(*i*,*j*) stored in location (*i*,*j*) can be defined as the occurrence frequency of a pair of pixels in a given distance and direction where the value of *f*(*x*_1_,*y*_1_) = *i* and the value of *f*(*x*_2_,*y*_2_) = *j*. A GLCM can be calculated for any distance, (*d = 1*,.*…*.,*n*) pixels in both directions, where (n) is the number of columns present in an image and over four angles (*θ)* where (*θ = 0°*, *45°*, *90°* and *135°)* [29, 47, 63]. Since all the healthy and dying cells varied in size, we used the GLCM in a novel way to provide a uniform input space for the MLP classifier. We achieved this by creating GLCMs from each of the cell’s gradient-magnitude maps. In addition, we performed this using gradient-direction maps also as a comparison. For the purpose of our research, the gradient-magnitude and gradient-direction values were rescaled to (N) grey levels (where N = 8, 16, 32 and 64), and GLCM was computed for each cell for all four angles. A GLCM was calculated for distance *d* (where *d = 1*, *2*,*…*, *15)*, for angles *θ* (where *θ = 0°*, *45°*, *90°* and *135°)* in both the right and left direction. The higher boundary value of distance (*d = 15*) was determined by the number of columns present in the smallest cell. Each GLCM was vectorised and used as input space to the perceptron and MLP networks. Three groups of datasets were created based on the number of GLCMs produced from each cell. The numbers of GLCM produced per cell were 16, 32, and 60, respectively. The three groups of created datasets were defined based on the number of GLCMs produced from each cell. For the three groups, the numbers of produced GLCMs from each cell were 16, 32, and 60. The number of GLCMs produced were calculated from the different angles and distances used (i.e., no. angles used × no. of distances used = Total no. of GLCM produced) in creating the GLCMs. For example, a total of four different angles and 15 different distances were considered while producing GLCMs for group three. Thus, a total of 60 (= 4×15) GLCMs are produced for group three. Similarly, four different angles and four different distances were considered for group one producing 16 (= 4×4) GLCMs from each cell. For group two, four angles and eight distances were considered and 32 (= 4×8) GLCMs were produced from each cell. For each group of data, four different sizes of GLCM (8×8, 16×16, 32×32, and 64×64) were calculated. Thus, a total of 12 GLCM datasets were created per gradient map. The GLCM size is determined on the power of 2, i.e., GLCM size = 2^*n*^, where n = 3,4,5, and 6. We did not consider the value *n = 1*, *2* as the resultant GLCM would be too small for our purpose. Similarly, the resultant GLCM for *n> 6* would be too big for our purpose.

### 2.3. Classification

#### 2.3.1. Single-layer perceptron model

The single-layer perceptron (SLP) artificial neural network functions such that, if inputs from two different but linearly separable classes are presented to it, the SLP algorithm converges and position a decision boundary between the classes [64]. Although the multilayer perceptron can solve decision problems with complicated, sophisticated boundaries [65, 66], single layer perceptron can only solve linear problems. In other words, we can use the single-layer perceptron as a linear predictor. In single-layer perceptrons, input signals {*x*_*k*_} are multiplied with a set of adjustable weight {*w*_*k*_} to generate an intermediate output (*y)* which results in a quantised binary output (*y*_*q*_). The output then compared to the desired binary output (*d*_*q*_) and generates the error (*e*_*q*_). The error is then used to modify the weights [64, 66]. If ***W****(n)* and ***X****(n)* are N-dimensional column vectors with the (*n)* is the discrete-time index, then:

$$W\left(n+1\right)=W\left(n\right)+\mu e_{q}\left(n\right)X\left(n\right)$$ (4)

$$e_{q}\left(n\right)=\;d_{q}\left(n\right)-\;y_{q}\left(n\right)$$ (5)

$$y\left(n\right)=\;W\left(n\right)X\left(n\right)$$ (6)

Where, *μ* is the learning rate. A 0.1 learning rate was used for the SLP classifier.

The SLP did not employ hard thresholding. Rather used a SLP as a linear predictor with a linear activation function of y = x. To test for linearity and if the data is linearly predictable.

#### 2.3.2. Multi-layer perceptron model

The Multi-Layer Perceptron (MLP) model is a class of ANN that consists of input neurons, hidden layers of nodes and output nodes. Typically, the input signal propagates from input to output through the networks on a layer by layer basis. The Back Propagation algorithm (BP) [67], a widely used training algorithm in MLP, was employed as the learning method in this paper. Here we employ the scaled conjugate gradient descent method [68]. Let’s consider a ‘two hidden layers MLP architecture’ where *h-1* denotes the input layer, *h* denotes the first hidden layer, *h+1* denotes the second hidden layer, and *h+2* denotes the output layer. The subscripts ’*i’*, *’j’*, *’k’*, and *’l’* represent the neuron numbers in layer *h-1*, *h*, *h+1*, and *h+2*, respectively. The output of the MLP can be defined as follows [69]:

$$y_{l}^{h+2}=f\left(\sum_{k=1}^{P}x_{k}^{h+1}w_{kl}^{h+1}\right)$$ (7)

Where, $x_{k}^{h+1}$ = *kth* input of second hidden layer *h+2*; $w_{kl}^{h+1}$ = weight from the *kth* neuron of the second hidden layer *h+1* to the *lth* neuron of the output layer *h+2*; $y_{l}^{h+2}$ = *lth* output neuron of the output layer h*+2; P* = total number of neurons in the second hidden layer; *f* = sigmoid activation function.

The working of the backpropagation algorithm can be described as follows:

If the network error *e* is the difference between actual output and desired output at layer *h+2*, then the corresponding weight update equation and the delta error signal $\delta_{l}^{h+2}$ can be defined as follows:

$$w_{kl}^{h+2\;\left(New\right)}=w_{kl}^{h+2\;\left(Old\right)}+\eta \delta_{l}^{h+2}y_{l}^{h+1}$$ (8)

$$\delta_{l}^{h+2}=\;y_{l}^{h+2}\left(1-\;y_{l}^{h+2}\right)e$$ (9)

Where, *η* = the learning rate of network training.

The weight update at the layer *h+1* and the corresponding error $\delta_{k}^{h+1}$ can be defined as follows:

$$w_{jk}^{h+1\;\left(New\right)}=w_{jk}^{h+1\;\left(Old\right)}+\;\eta \delta_{k}^{h+1}y_{j}^{h}$$ (10)

$$\delta_{k}^{h+1}=\;y_{k}^{h+1}\left(1-\;y_{k}^{h+1}\right)\sum_{k=1}^{P}\left(\delta_{l}^{h+2}w_{kl}^{h+2}\right)$$ (11)

Where, *P* = total number of neurons in the second hidden layer.

The weight update at the layer *h* and the corresponding error $\delta_{j}^{h}$ can be defined as follows:

$$w_{ij}^{h\;\left(New\right)}=w_{ij}^{h\;\left(Old\right)}+\;\eta \delta_{j}^{h}x_{i}^{h-1}$$ (12)

$$\delta_{j}^{h}=\;y_{j}^{h}\left(1-\;y_{j}^{h}\right)\sum_{j=1}^{N}\left(\delta_{k}^{h+1}w_{jk}^{h+1}\right)$$ (13)

Where, *N* = total number of neurons in the first hidden layer; and $x_{i}^{h-1}$ = the ith input in the input layer.

For the purpose of our study, the datasets were used as the input space to a one hidden layer MLP and then as the input space to a two hidden layer MLP model. The number of neurons in each hidden layer was determined by the trial-and-error method. The number of hidden neurons ranged from 2/3^rd^ of the neurons in the input-layer to the total amount of neurons in the input-layer. From the results of the above-mentioned trials and to avoid overfitting, the optimal number of hidden neurons was determined to be half of its input neuron number. Thus, the hidden neuron numbers used are as follows.

1. For the 8×8 GLCM dataset, 1^st^ hidden layer = 32 and 2^nd^ hidden layer = 16.
2. For the 16×16 GLCM dataset, 1^st^ hidden layer = 128 and 2^nd^ hidden layer = 64.
3. For the 32×32 GLCM dataset, 1^st^ hidden layer = 512 and 2^nd^ hidden layer = 256.
4. For the 64×64 GLCM dataset, 1^st^ hidden layer = 2048 and 2^nd^ hidden layer = 1024.

The goal of all ANN designs was to determine the simplest architecture that would provide the highest performance. All the models were developed using the (MATLAB © programming) environment.

The main advantage of shallow learning MLP approaches is that they can provide an architecture that is of low complexity requiring less time to converge and minimal data for training. The drawback of MLP shallow learning approaches is that, in general, no performance gain above to 2 hidden layers of processing is obtained, which could serve to limit subtle nonlinear feature recognition. The advantages of deep learning approaches are that many hidden layers can be realised, permitting subtle nonlinear features to be recognised, which can serve to improve performance over shallow learning. However, the disadvantages of deep learning approaches are large architectural complexity requiring very large amounts of data for convergence and large training times for convergence in comparison to MLP shallow learning approaches.

It should be noted that the goal for any ANN approach is to select the approach that has the highest performance but also whose architecture is as low in complexity as possible. Low architectural complexity is often overlooked since the advent of deep learning methods due to the gain to be made in performance. However, this does not mean that shallow learning approaches are old or redundant. Our reasoning for solving this problem was to first determine if the data was linear and hence, could be predicted with a linear predictor (hence an SLP model approach–which is often overlooked in ANN prediction per see) [70]. Then we determine if a shallow learning approach was sufficient to predict the data. In this article, we examined the 1 and 2 hidden layer MLP. The reason for this is that it would provide further insight into the level of nonlinear complexity within a shallow learning scheme. (Namely, good prediction with a 1 hidden MLP would demonstrate that the XOR problem is not required to be solved, thus inferring low nonlinear complexity exists for a shallow learning solution and good prediction with 2 hidden layers infers that the XOR problem is required and infers that high nonlinear complexity exists for a shallow learning solution) [71]. Since we found that the performance of our GD-GLCM training method was almost optimal already, we did not feel that it was necessary to compare it to new methods as there is no significant performance gain to be made. However, if we had found it not to be optimal, then deep learning approaches would be used to scavenge the data for further structure, but this would require much larger amounts of data and increase training times due to the large architectural complexity of a deep network model.

### 2.4. K-fold cross-validation & classification performance measurements

We employed a k-fold cross-validation method to validate our classification performance where the k = 5. All the cells obtained from the 17 sheep were initially randomized to create a single image dataset. This dataset was then divided into k = 5 equal parts. Each of the five parts was then selected one at a time as the validation dataset, while the other four parts were selected as the training dataset. To ensure that there was no data leakage, the whole data set was split 80:20 as training and validation sets, respectively. Bar plots were constructed in section 3, to highlight the 5-fold cross-validation of the means of the sensitivity, selectivity and accuracy and 5-fold cross-validation of the standard deviation as error bars.

Since we trained on portions of cells in all images and validated on other portions of cells in all images then, the network may be aware of the dependency of features across all sheep. A future improvement of this would be to train on all sheep but one to provide an independent validation set.

The performance of the proposed algorithm was assessed with parameters recall or sensitivity (14), precision or selectivity (15), and accuracy(16).

Sensitivity=TP/(TP+FN)×100 (14)

Selectivity=TP/(TP+FP)×100 (15)

$$Accuracy=\left(TP\;+\;TN\right)\;/\;\left(TP\;+\;TN\;+\;FP\;+\;FN\right)\;\times \;100$$ (16)

Where, (TP = True Positive; Tn = True Negative; FP = False Positive and FN = False Negative).

We have plotted a ROC curve for each class, i.e., a total of two ROC curves for each classification performance. The reason we have two ROC curves is to show ROC curves for both healthy and dying classes of cell. We designed our study as a multi-class, multi-label classification problem. For the multi-class, multi-label classification, it is standard practice to plot one ROC curve for each class [72].

The reason for defining our study as a multi-class multi-label classification problem instead of a binary classification problem is as follows. In our research, we aimed to find out not only the dying cells, but also to see how much each cell belongs into each class. The knowledge of this information impacts our future research.

To summarise the ROC curves, we calculated the area under the curve (AUC) of ROC.

## 3. Results

### 3.1. Standard methodology

The standard method involved creating GLCMs from the grey-level image values directly. Texture Analysis was then performed by calculating the ten main Haralick’s texture parameters (contrast, energy, entropy, homogeneity, variance, sum average, sum variance, sum entropy, difference variance, difference entropy) from the GLCMs [73]. The 10 Haralick texture parameters were then passed as a vector to the input space of an ANN classifier. For the standard methodology, a two hidden layer MLP was used. Results of the standard method are displayed for comparison, besides the results of the proposed GD-GLCM Image training method now described.

### 3.2. Novel gradient direction, grey level co-occurrence matrix image training

**Fig 3:**
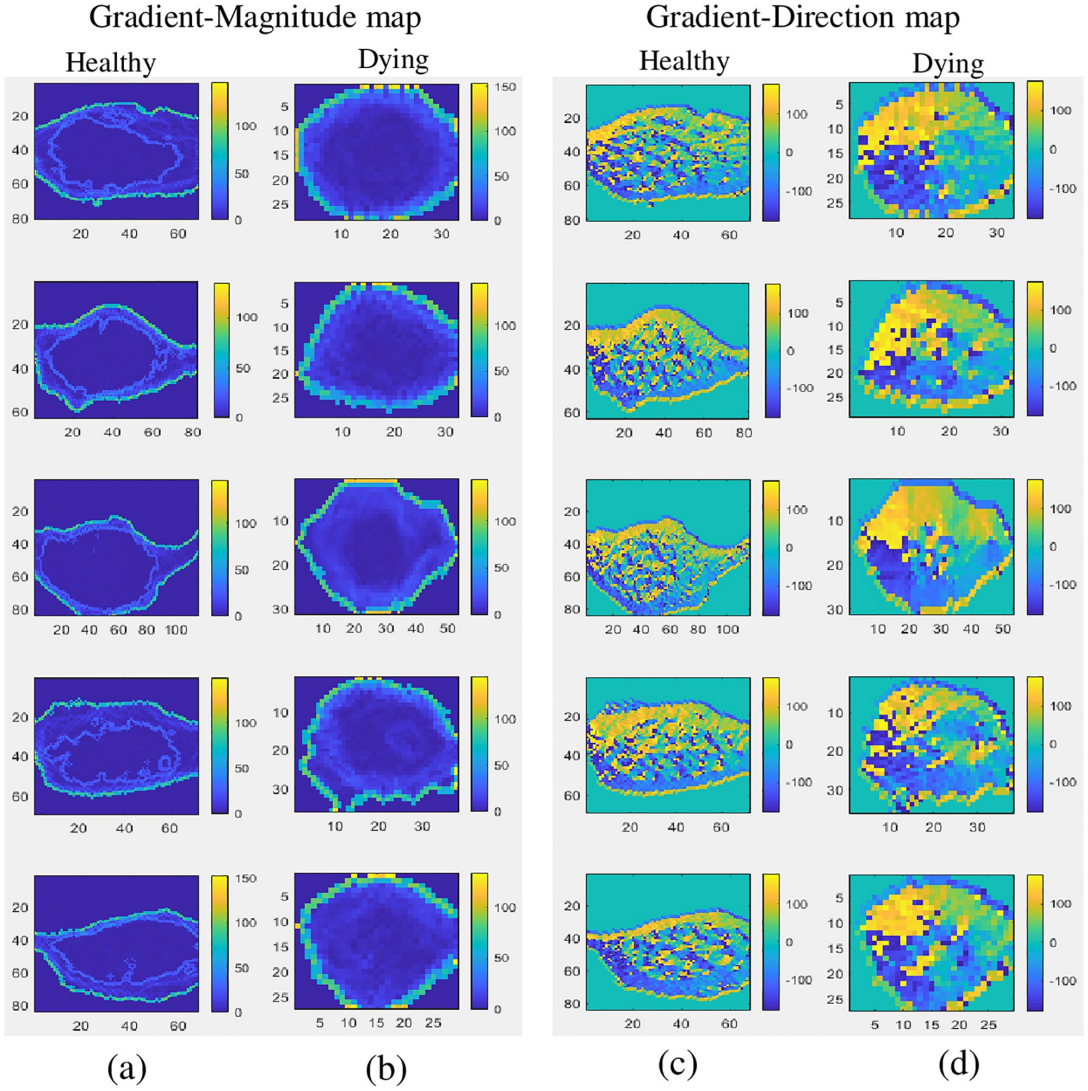
Colour coded gradient-magnitude and gradient-direction maps of healthy and dying cells (a) Gradient-magnitude maps of 5 healthy cells, (b) gradient-magnitude maps of 5 dying cells, (c) gradient-direction maps of 5 healthy cells and (d) gradient-direction maps of 5 dying cells.

Here we present a ’Gradient Direction, Grey level Co-occurrence Matrix’ (GD-GLCM) image training method which simplifies the standard training methodology used to classify cell images. This is by determining the GLCM of the gradient direction (GD) of a cell image followed by direct passing to an ANN in the form of a Multilayer Perceptron (MLP). Hence, avoiding all texture feature analysis steps. In addition, we create a ’Gradient Magnitude, Grey level Co-occurrence Matrix’ (GM-GLCM) image training method which employs gradient magnitude for comparison. Fig 3 shows the colour coded gradient-magnitude and gradient-direction maps of the healthy and dying cells.

**Fig 4:**
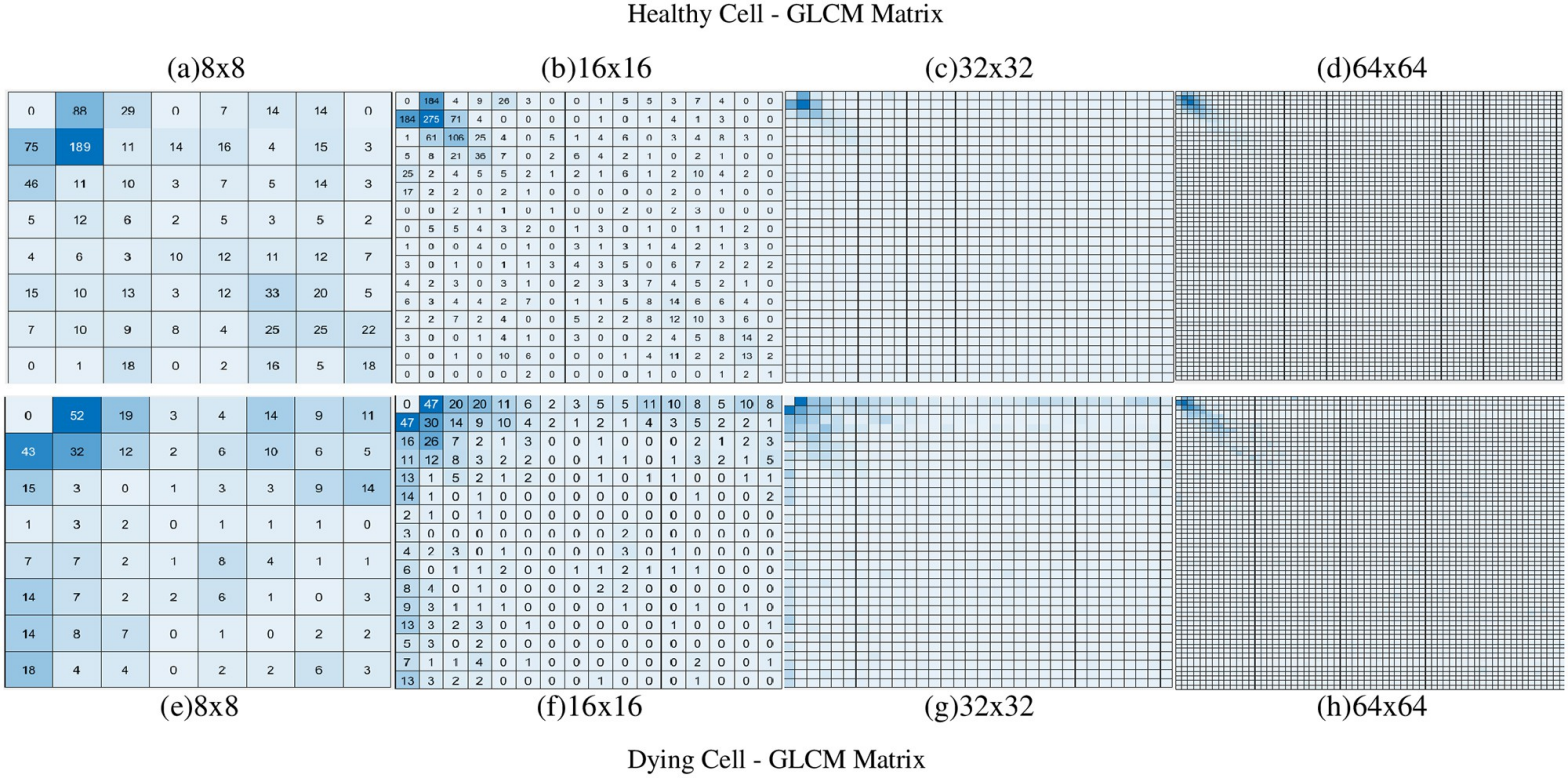
Example of the GLCM heatmap derived from GM values. GM values of a healthy cell (a, b, c, d) and a dying cell (e, f, g, h) when generating 16 GLCMs/cell.

**Fig 5:**
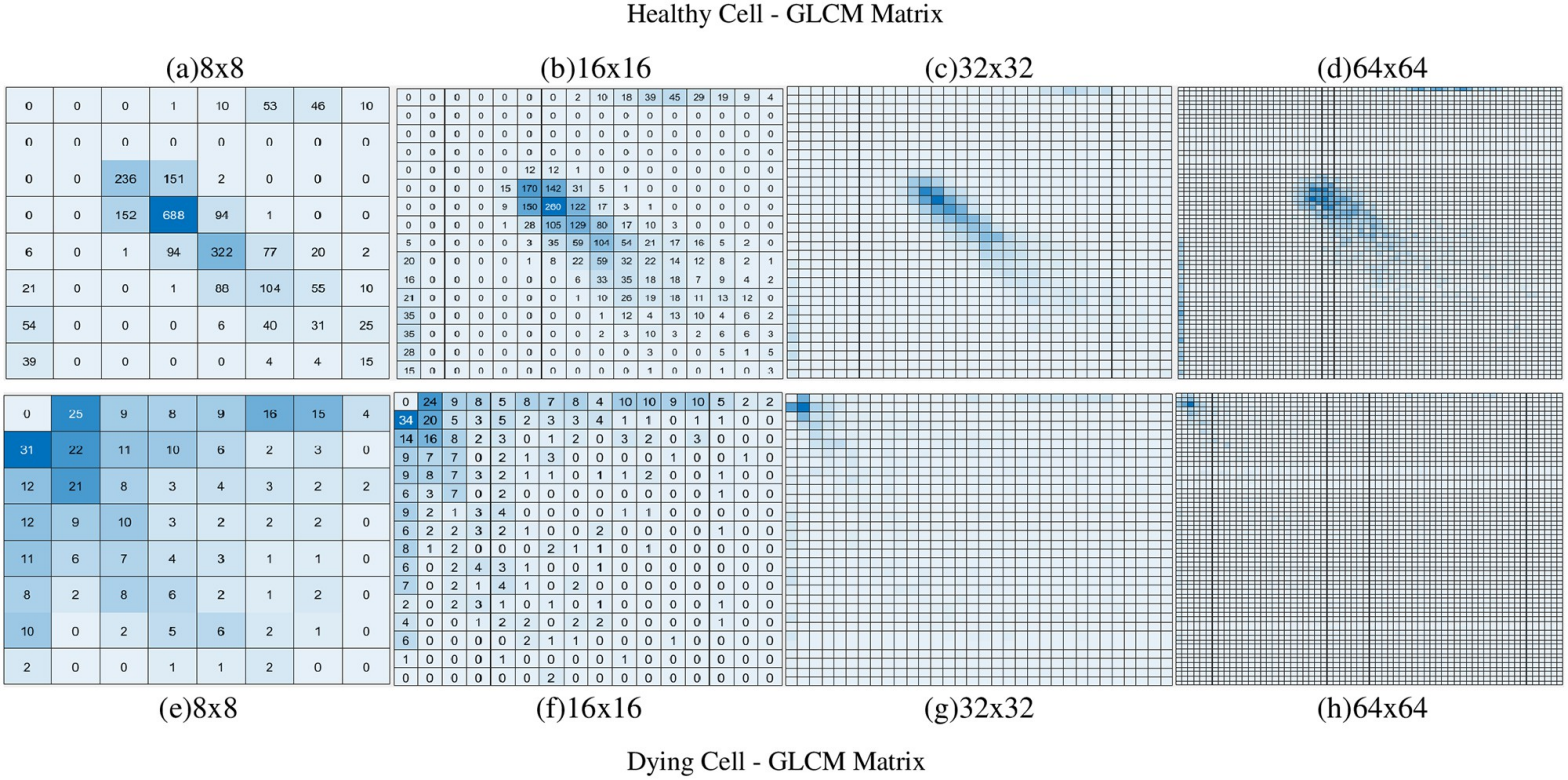
Example of the GLCM heatmap derived from GD values. GD values of a healthy cell (a, b, c, d) and a dying cell (e, f, g, h) when generating 16 GLCMs/cell.

It was observed, from Fig 3, that the GM maps and GD maps provided consistent and repeatable patterns for healthy neurons that were notably different to the dying neurons. Using the GLCM, we then transformed the GM and GD maps in order to provide novel uniform datasets as input to the SLP and MLP networks. We created different sized GLCMs (8×8, 16×16, 32×32 and 64×64) to assess the performance of the SLP and MLP networks. Heatmaps of the GLCMs are shown in Figs 4 and 5 below.

From Fig 4, it can be observed that the GLCM heatmaps between the healthy and dying cells were very similar when the GLCMs were derived from GM values. For 32×32 (Fig 4c and 4g) and 64×64 (Fig 4d and 4h), no major difference between healthy and dying cells can be visually identified. However, the heatmaps from Fig 5 show that the GLCMs are quite markedly different for healthy and dying cells.

**Fig 6:**
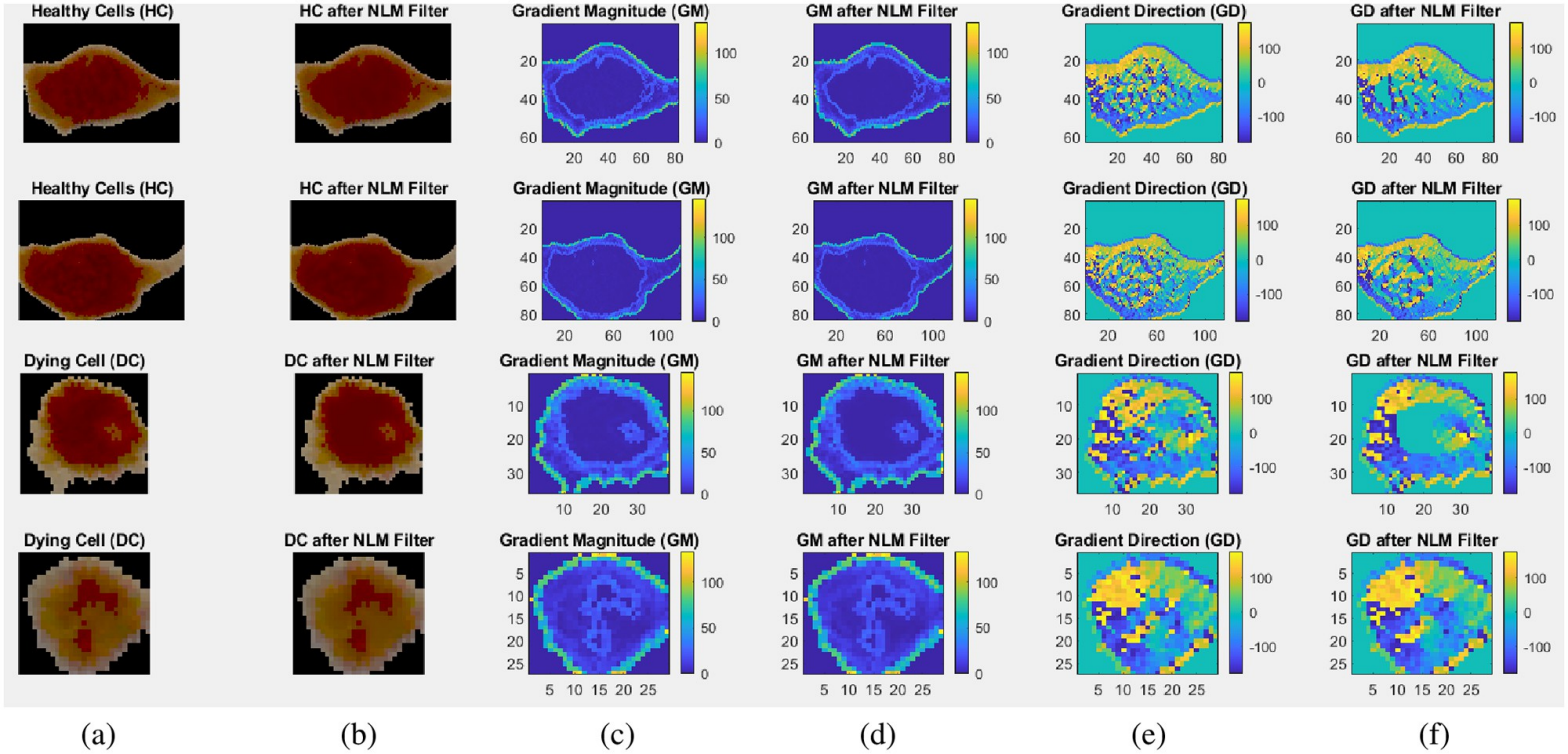
Healthy and dying cells with their GM and GD colourmaps in pre and post NLM filtered states. (a) shows two healthy and two dying cells before application of NLM filter, (b) shows those cells after application of NLM filter, (c) shows GM colourmaps of those cells before NLM filter application, (d) shows GM colourmaps of those cells after NLM filter application. (e) shows GD colourmaps of those cells before NLM filter application, and (f) shows GD colourmaps of those cells after NLM filter application.

Furthermore, in Fig 6, we demonstrate how a non-local means (NLM) smoothing filter whilst providing noise reduction in the post NLM cell images (for both healthy and dying cells) serves to remove some of the nonlinear detail in the GD and GM images. No differences could be observed in the colour map images of GM in the pre and post filtered state, a blurriness could be observed in the post filtered GD images, as shown in Fig 6. Such blurriness would represent a loss of the textural and nonlinear information for classification. Thus, it was decided not to employ a smoothing filter. In addition, noise in the data would serve to improve generalisation of the MLP (like the technique of noise injection [69]) especially when the data was recast in many ways considering several angles and several distance between the pixels while calculating the GLCM. When we did trial out a smoothing filter, we found that it created problems with our segmentation process. This was because the cell boundaries were already hard to determine in the raw images; thus, using a smoothing filter made this more difficult for the aforementioned reasons.

**Fig 7:**
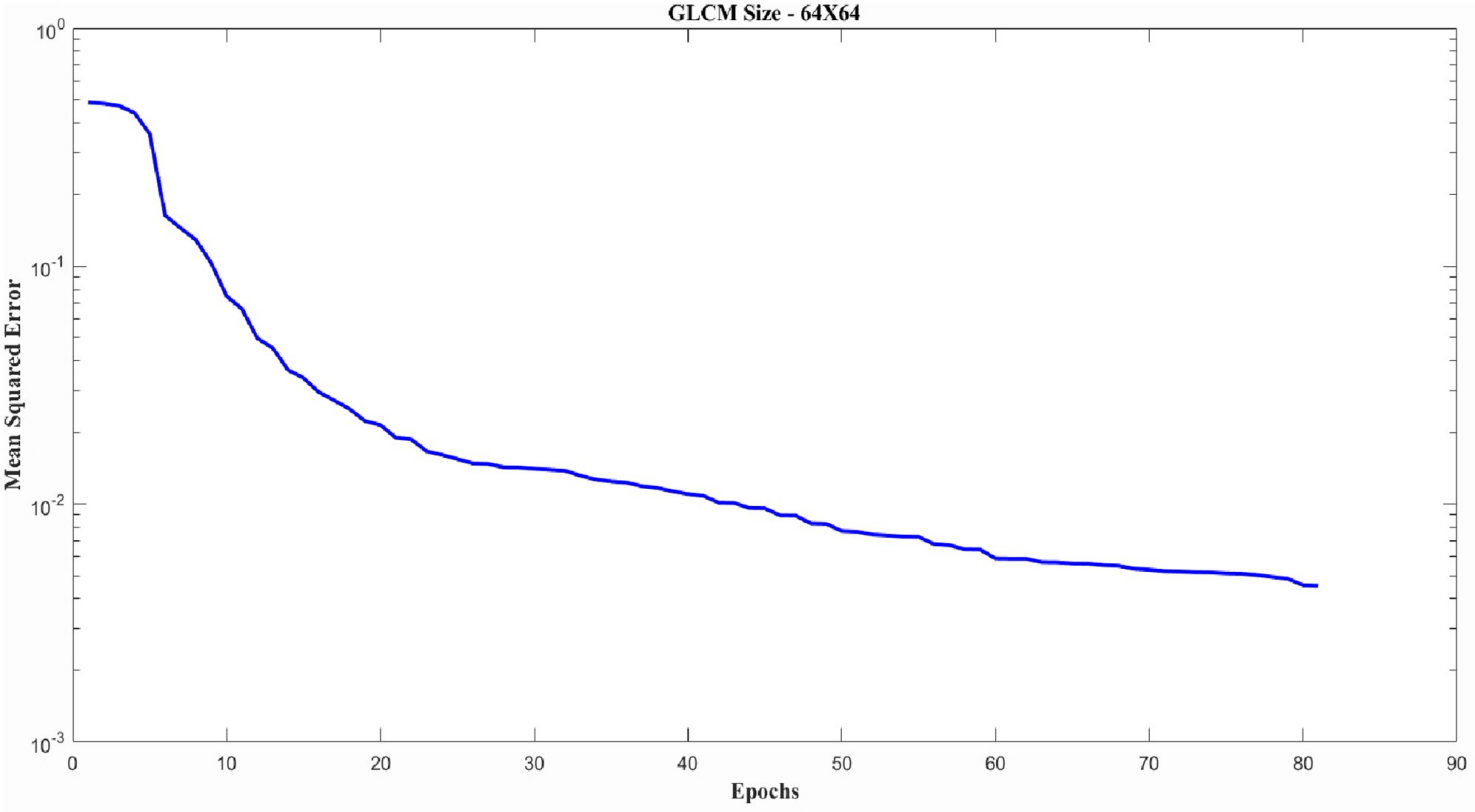
Typical error curve for training GD input datasets where GLCM size is 64×64 for MLP classifier.

As an example, a typical error curve for training the GD input datasets with GLCM size 64×64 for an MLP classifier is provided in Fig 7 below. All the training error curves were found to be similar to the curve shown in Fig 7 and do not exhibit over-fitting (The only except to this were a few classification failures (as shown in Fig 9 ROC curves and Fig 10 AUC plot) which were identified for the SLP classifier.

Moreover, we have taken precautions to avoid overfitting. They are as follows.

1. We also used K-fold cross-validation to reduce the chance of overfitting.
2. We also used early stopping in case of validation failure (max validation fails set to 5) to avoid the risk of overfitting.

#### 3.2.1. Single-layer perceptron (SLP) classification results

**Fig 8:**
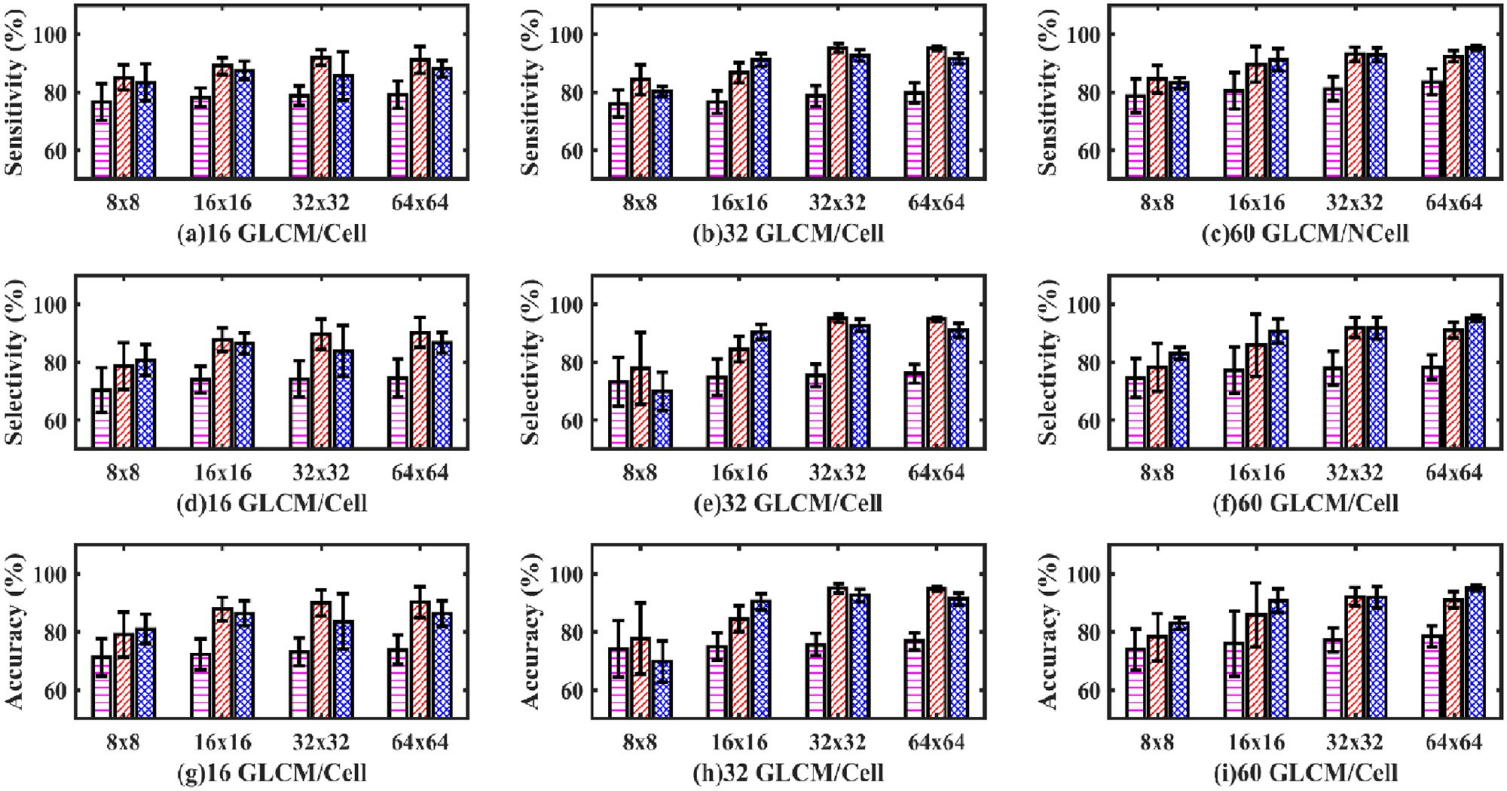
Sensitivity, selectivity, and accuracy bar plots of an SLP classifier. Sensitivity (a, b, c), selectivity (d, e, f) and accuracy (g, h, i) bar plot of an SLP classifier. (Magenta dashed bar, standard method; Red slashed bar, GM-GLCM image training; Blue crossed bar, GD-GLCM image training method. The x-axis defines the size of GLCM arrays passed to the SLP network (namely, 8×8, 16×16, 32×32 and 64×64). The caption label defines how many GLCMs arrays were passed to the SLP network for training/cell (namely 16,32 or 60).

As mentioned previously, for each group, four different sizes of the GLCMs were calculated. The four matrix sizes selected were 8×8, 16×16, 32×32 and 64×64, respectively. In addition, we also trained the networks on different amounts of GLCMs/cell to determine the optimum training of the networks. Hence, the networks were trained on three different amounts of GLCMs/cell. (These were 16 GLCMs created/cell, 32 GLCMs created/cell, and 60 GLCMs created/cell). We reported sensitivity, selectivity, and accuracy (Fig 8) for all the 12 different combinations of the aforementioned datasets when passed to a single-layer perceptron neural network classifier.

Consistent trends were observed for the sensitivity (a, b, c), selectivity (d, e, f) and accuracy (g, h, i) bar plots of Fig 8, showing how increasing performance occurred for increasing GLCM size, which plateaued when the GLCM size went from 32×32 to 64×64. It can be seen that optimising for the best performance with the most reduced input space for the network leads to 32 GLCM/cell. It can also be observed on the whole that the standard method had lower sensitivity, selectivity and accuracy than the GD-GLCM and GM-GLCM image training methods.

**Fig 9:**
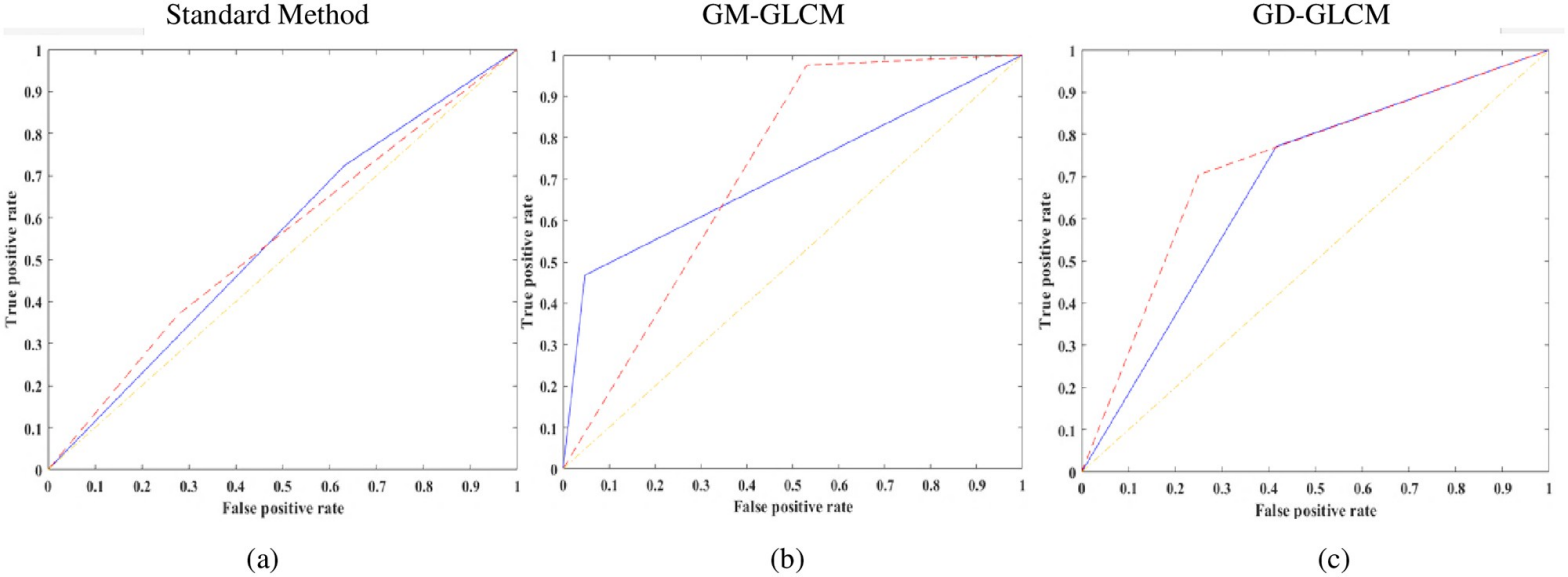
ROC curves for healthy and dying cells of an SLP classifier. Average ROC curves for healthy and dying cells using the optimised 32 GLCMs/cell category and GLCM size of 64×64 for the SLP classifier (Red dashed line–healthy cells; Blue solid line–dying cells). (a) using standard method with 64×64 GLCM size, (b) using GM-GLCM with 64×64 GLCM size, (c) using GD-GLCM with 64×64 GLCM size.

**Fig 10:**
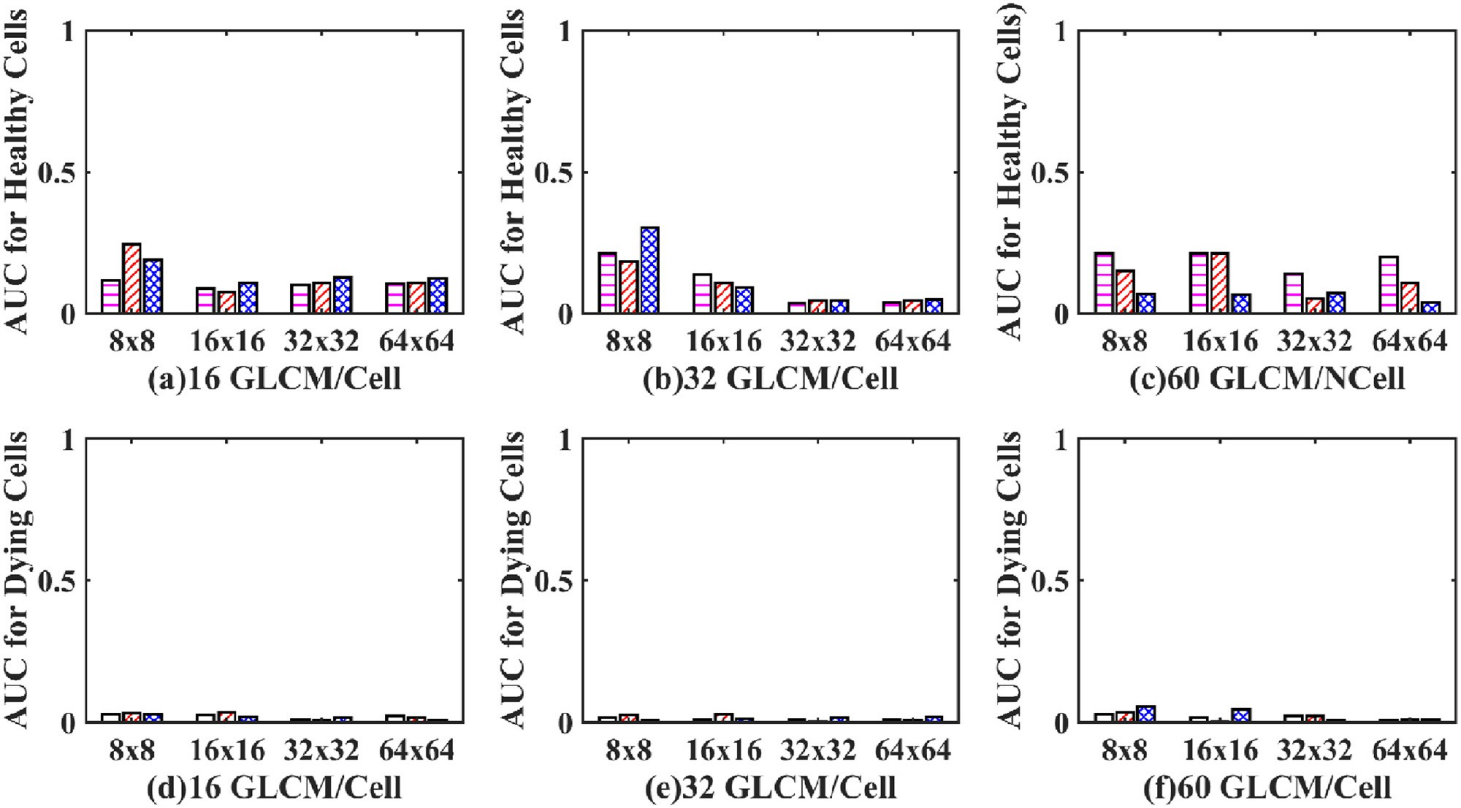
AUC for healthy and dying cells bar plot of an SLP classifier. (Magenta dashed bar, standard method; Red dashed bar, GM-GLCM method; Blue crossed bar, GD-GLCM method).

Receiver operating curves (ROC, Fig 9) were plotted, and the area under the curve (AUC) of the ROC curves, Fig 10, were calculated to validate performance for both the healthy and dying cell categories to verify the training performance.

Fig 9 shows an example of average ROC curves for both the healthy and dying cells of the optimised 32 GLCMs/cell category and GLCM sizes of 64×64 for the SLP classifier. It can be seen that all the curves exhibit a low true-positive rate or high false-positive rate, or both where the standard method provides a much lower ROC curve compared to GD-GLCM and GM-GLCM image training methods.

From the ROC curves of Fig 9, the AUC was derived in Fig 10. It can be observed that despite having good sensitivity, selectivity, and accuracy values, of Fig 8, the AUC values for both healthy and dying cells are discouragingly small for the SLP classifier.

#### 3.2.2. Multi-layer perceptron (MLP) with one hidden layer classification results

**Fig 11:**
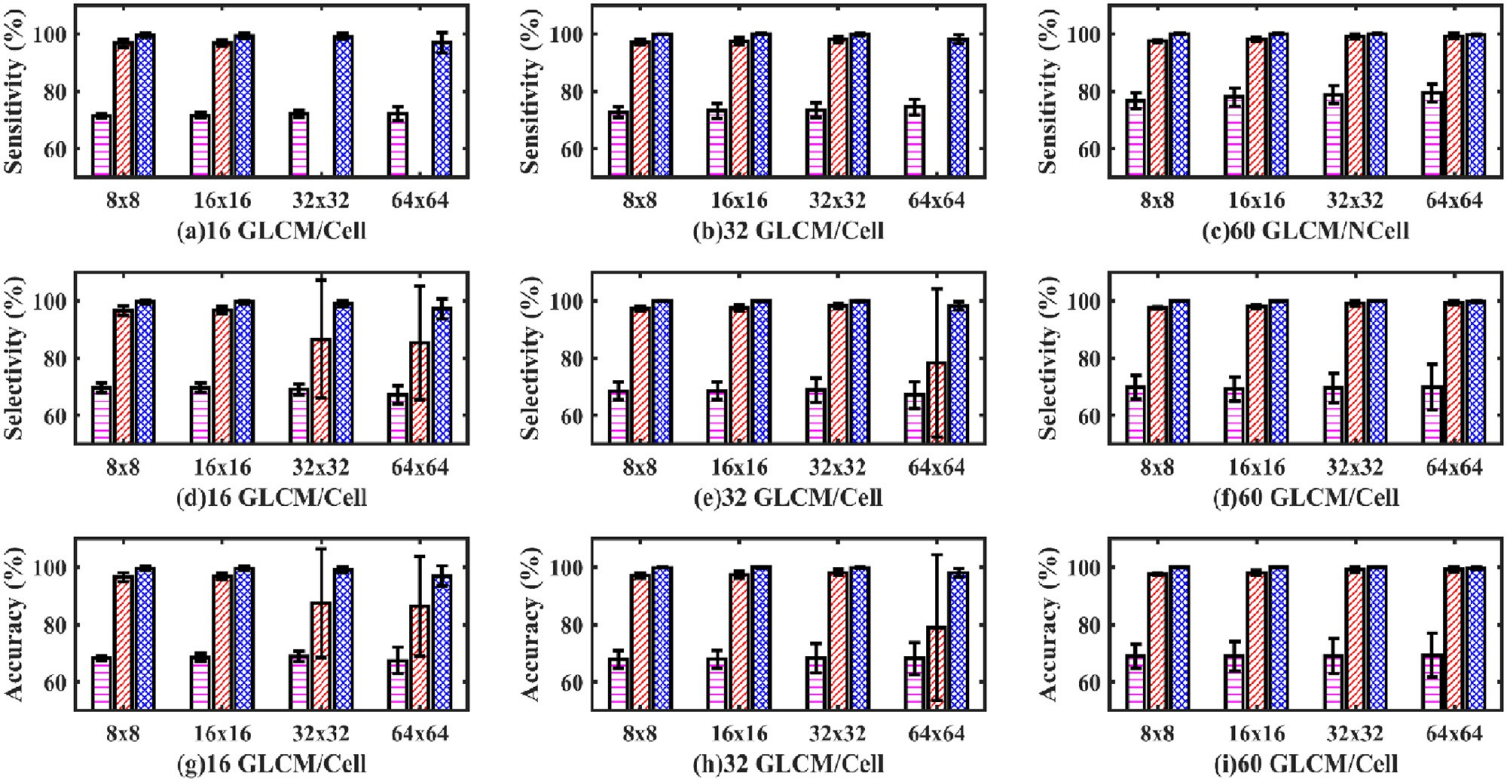
Sensitivity, selectivity, and accuracy bar plots of one hidden layer MLP classifier. Sensitivity (a, b, c), selectivity (d, e, f) and accuracy (g, h, i) bar plot of one hidden layer MLP classifier. (Magenta dashed bar, standard method; Red slashed bar, GM-GLC method; Blue crossed bar, GD-GLCM method). The x-axis defines the size of GLCM arrays passed to the SLP network (namely, 8×8, 16×16, 32×32 and 64×64). The caption label defines how many GLCMs arrays were passed to the SLP network for training/cell (namely 16,32 or 60).

Next, an MLP artificial neural network model with one hidden layer was used to classify healthy from dying cortical neurons of a fetal sheep exposed to global ischemia. Fig 11 shows the sensitivity (a, b, c), selectivity (d, e, f), and accuracy (g, h, i) bar plot for the one hidden layer MLP model.

It can be observed that for all plots of Fig 11(a)–11(i) that the GD-GLCM image training method remains consistently stable and high with accuracies of mean range (97.04% ±3.5% to 99.65% ± 0.33%) over the GM-GLCM method (86.37% ±17.45% to 99.21% ± 0.86%). It should be noted that the sensitivity could not be calculated for the 32×32 and 64×64 GM-GLCM methods of Fig 11(a) due to classification failure on those two cases. It was observed that the standard method results were poor mean range (67.44% ±4.64% to 69.3% ± 7.7%) and consistently less in comparison to the GM-GLCM and GD-GLCM image training methods.

**Fig 12:**
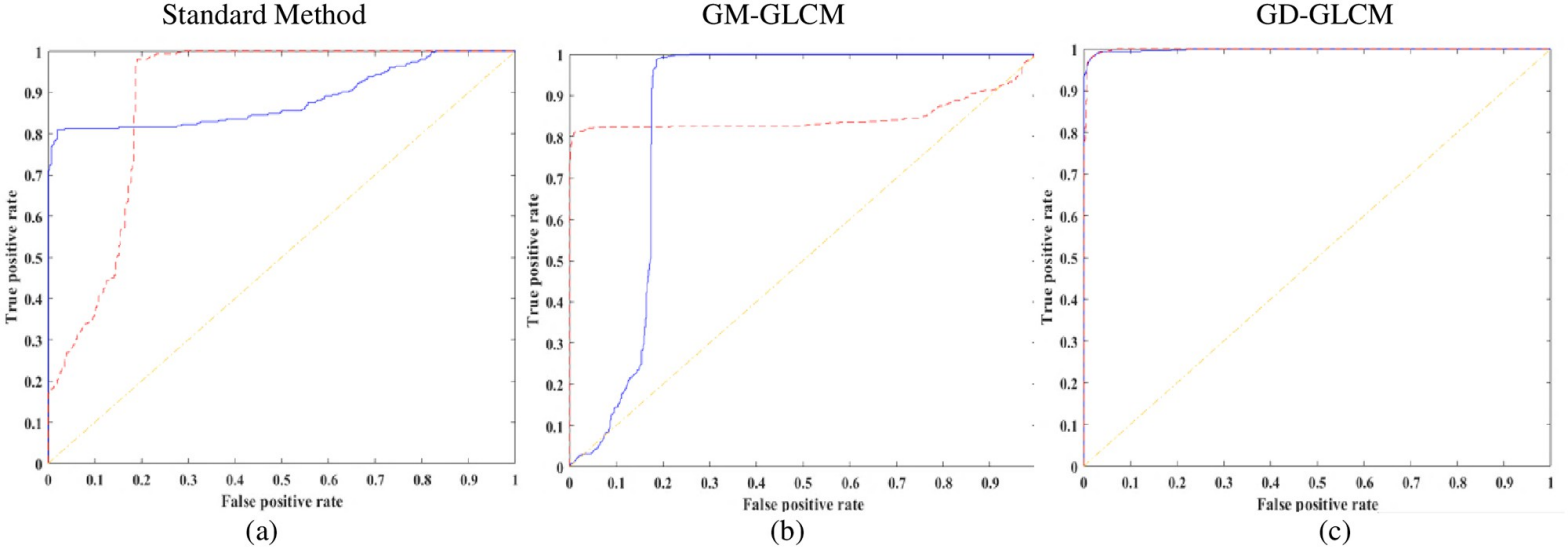
ROC curves for healthy and dying cells of one hidden layer MLP classifier. Average ROC curves for healthy and dying cells using the optimised 32 GLCMs/cell category and GLCM size of 64×64 for the one hidden layer MLP classifier (Red dashed line–healthy cells; Blue solid line–dying cells). (a) using standard method with 64×64 GLCM size, (b) using GM-GLCM with 64×64 GLCM size, (c) using GD-GLCM with 64×64 GLCM size.

The average ROC curves in Fig 12, show inferior performance for the 32 GLCM/neuron group when the matrix size is 64×64 when using the GM-GLCM method (Fig 12b). In contrast, for the same group and same GLCM size, using the GD-GLCM image training method (Fig 10c) achieved good performance. Although, the GM-GLCM and the standard method achieved similar results, the GD-GLCM methods achieved better performance than both of them.

**Fig 13:**
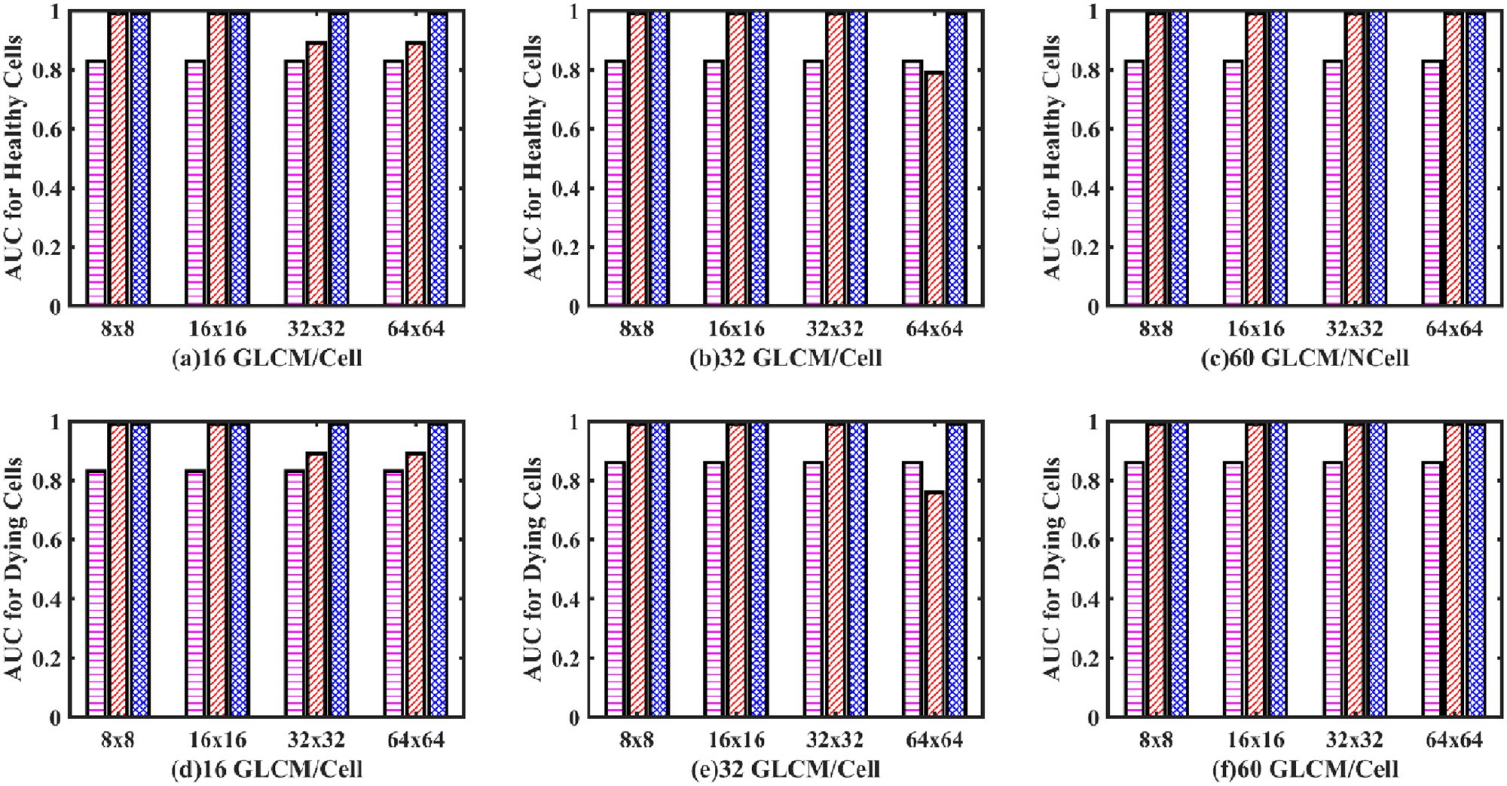
AUC for healthy and dying cells bar plot of one hidden layer MLP classifier. (Magenta dashed bar, standard method; Red dashed bar, GM-GLCM method; Blue crossed bar, GD-GLCM method).

From the ROC curves of Fig 12, the AUC was derived in Fig 13. It can be observed from Fig 13 that the AUC values for both the healthy and dying cells for the GD-GLCM image training method were excellent at 99.96%. Regarding the GM-GLCM method for both healthy and dying cells, Fig 13a shows a drop in AUC values at 32×32 and 64×64 GLCM to 89.3% when 16 GLCM/cell are passed to the network. This was observed to improve to 99% for 32×32 GLCM sizes when 32 GLCM/cell were passed to the network and improved further to 99% for 64×64 GLCM sizes when 60 GLCM/cell were passed to the network. Thus, it was found that increasing the number of GLCM/cell to the network increased the AUC for the GM-GLCM method. It was observed that the AUC values for both the healthy and dying cells using the standard method were significantly lower at ~ 86% in comparison to the GM-GLCM and GD-GLCM image training methods.

#### 3.2.3. Multi-layer perceptron (MLP) with two hidden layer classification results

**Fig 14:**
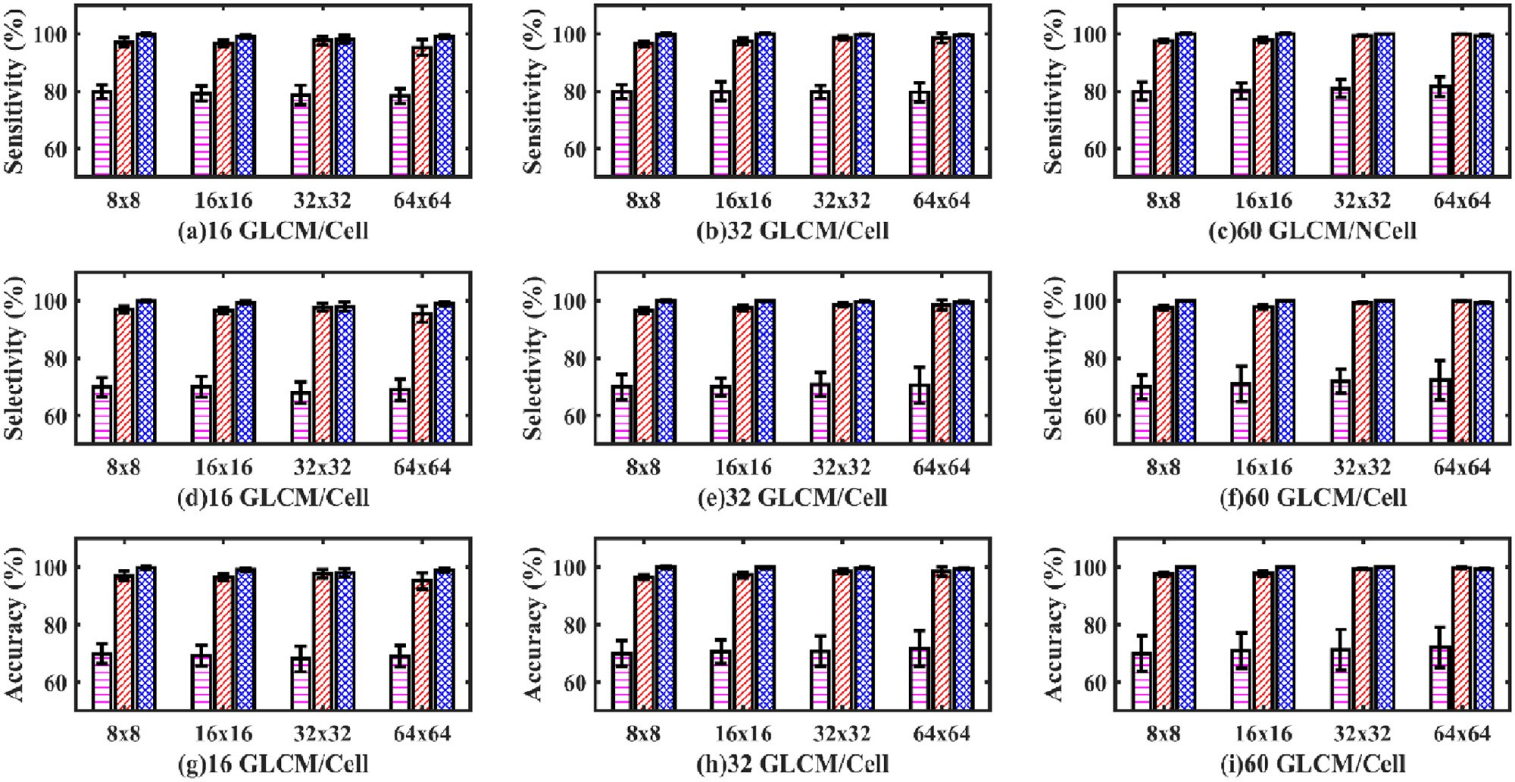
Sensitivity, selectivity, and accuracy bar plots of two hidden layer MLP classifier. Sensitivity (a, b, c), selectivity (d, e, f) and accuracy (g, h, i) bar plot of two hidden layer MLP classifier. (Magenta dashed bar, standard method; Red slashed bar, GM-GLCM method; Blue crossed bar, GD-GLCM method). The x-axis defines the size of GLCM arrays passed to the SLP network (namely, 8×8, 16×16, 32×32 and 64×64). The caption label defines how many GLCMs arrays were passed to the SLP network for training/cell (namely 16, 32 or 60).

Finally, an MLP neural network model with two hidden layers was used to classify healthy from dying cortical neurons. Fig 14 shows the sensitivity (a, b, c), selectivity (d, e, f), and accuracy (g, h, i) bar plot for the two hidden layer MLP model for the three methods.

It can be observed that for all plots of Fig 14(a)–14(i) that the GD-GLCM image training method provided consistently stable and high with accuracies of mean range (98.03% ±1.5% to 99.96% ± 0.09%). For the GM-GLCM method, slightly lower but still consistently high and stable accuracy now occurred of mean range (95.23% ±2.8% to 99.87% ± 0.81%). It was observed that the standard method performed significantly lower with mean range (69.04% ±3.37% to 72.1% ± 7.3%) in comparison to the GM-GLCM and GD-GLCM methods.

**Fig 15:**
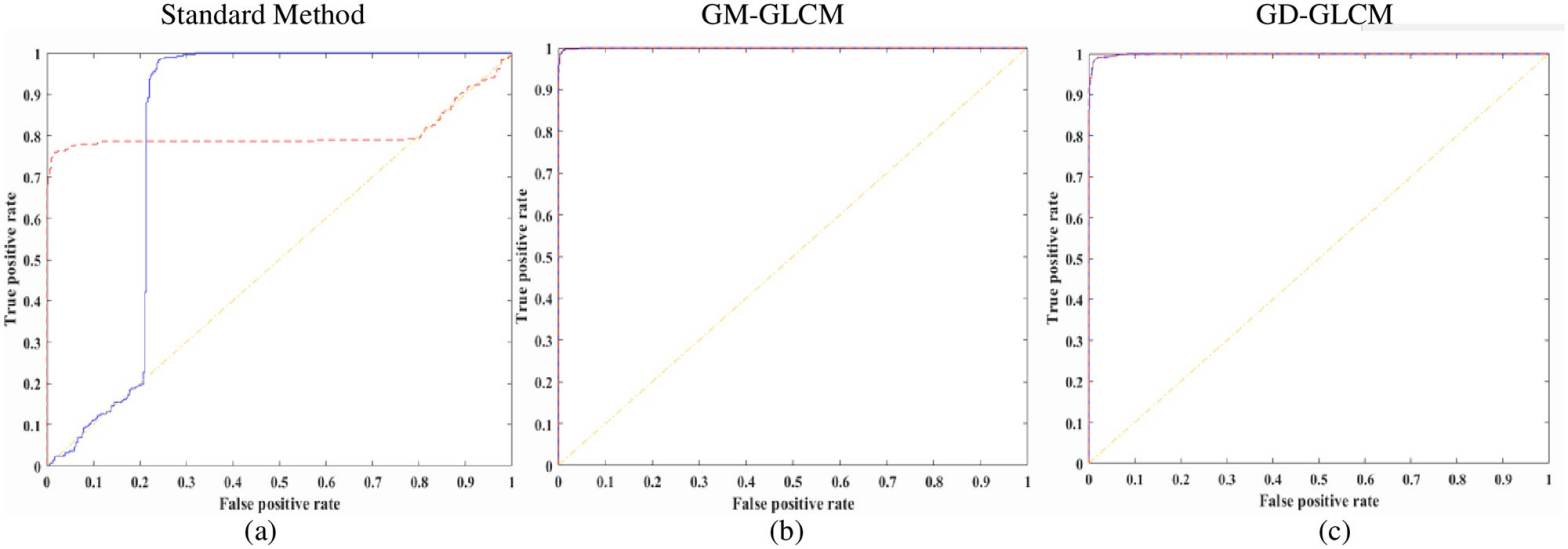
ROC curves for healthy and dying cells of two hidden layer MLP classifier. Average ROC curves for healthy and dying cells using the optimised 32 GLCMs/cell category and GLCM size of 64×64 for the two hidden layer MLP classifier (Red dashed line–healthy cells; Blue solid line–dying cells). (a) using standard method with 64×64 GLCM size, (b) using GM-GLCM with 64×64 GLCM size, (c) using GD-GLCM with 64×64 GLCM size.

Fig 15 shows excellent performance with ROC curves for both the GD-GLCM and GM-GLCM method (Fig 15b and 15c). It could be observed that the ROC curve for the standard method was poorer than the GM-GLCM and GD-GLCM image training methods.

**Fig 16:**
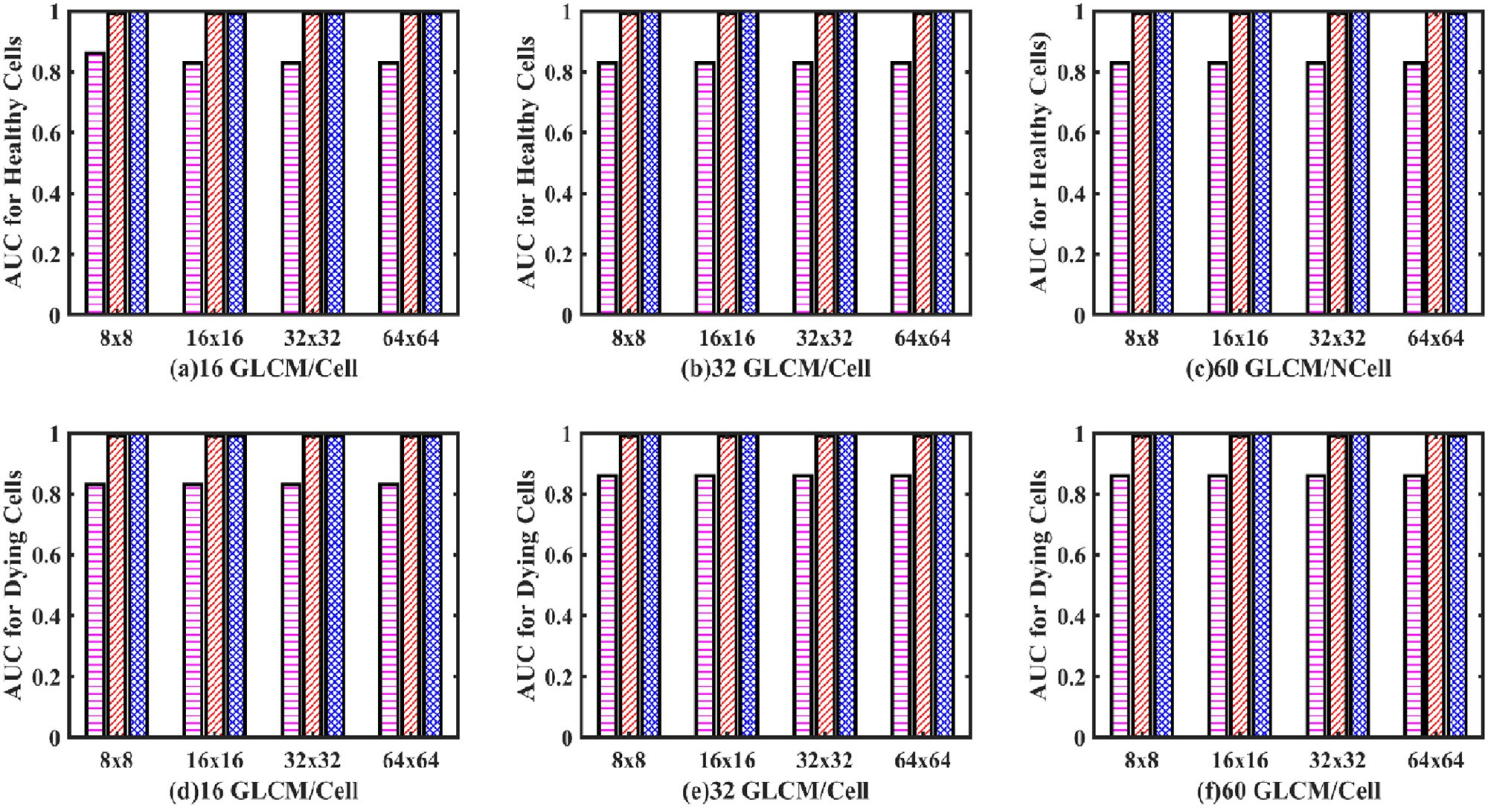
AUC for healthy and dying cells bar plot of two hidden layer MLP classifier. (Magenta dashed bar, standard method; Red dashed bar, GM-GLCM method; Blue crossed bar, GD-GLCM method).

Fig 16 shows excellent AUC values for all groups of both the healthy and dying cells for both GM-GLCM and GD-CLCM methods obtaining an AUC of 99.96% for the GD-GLCM image training method. It was observed that the standard method produced much a lower AUC of 86% compared to both GM-GLCM and GD-GLCM training methods. Thus, indicating the most successful classification between healthy and dying cells using a two hidden layer MLP network.

## 4. Discussion and conclusion

In this article, we presented a ’Gradient Direction, Grey level Co-occurrence Matrix’ (GD-GLCM) image training method which simplifies the standard training methodology used to classify healthy and dying cortical cells in hypoxic-ischemic fetal sheep brain slice images. This was by determining the GLCM of the gradient direction (GD) of a cell image followed by direct passing to an ANN in the form of a Multilayer Perceptron (MLP). Hence, avoiding all texture feature analysis steps that are required in the standard method. We compared the performance of a perceptron, 1-layer and 2-layer MLP when standard training was performed, on GD-GLCM image training was performed as well as comparing the performance using gradient magnitude instead of gradient direction.

It was found for the single-layer perceptron (SLP) model that the accuracy, precision and sensitivity values were relatively high for some cases (especially for the GD–GLCM image training method). SLP produces accuracy in the range of (71.3 ± 6.41% to 78.51 ± 3.74%) for the standard method; (79.1 ± 7.68% to 90.96 ± 2.77) for the GM–GLCM method and (80.93 ± 5.11% to 95.17 ± 1.08%) for the GD–GLCM method. However, the ROC curves and AUC values for identifying dying and healthy cells told a very different story. The ROC curves showed that when the true-positive rate was low for the dying neurons, then the false-positive rate became high for healthy neurons and vice versa. From this information, we can infer that the classifier detects most of the neurons as one class (either dying or healthy neurons), consequently proving that the image data is most likely nonlinear and that a linear SLP model is not appropriate for this class of problem. This was further emphasised by the poor AUC values (< 0.35 for healthy cells and was less < 0.1 for dying cells) that indicates classification failure.

It was found that for a one hidden layer MLP, that accuracies in the range of (67.44% ±4.64% to 69.3% ± 7.7%) could be achieved for the standard method; (86.37% ±17.45% to 99.21% ± 0.86%) for the GM–GLCM method and (97.04% ±3.5% to 99.65% ± 0.33%) for the GD–GLCM method. Thus, the standard method provided a far poorer accuracy than either of the GM–GLCM or GD–GLCM image training methods. Overall, the GD–GLCM image training method was found to have the most consistent and highest accuracy. A curious observation was made of the GM–GLCM method. It was found that a sudden drop in accuracy values and an increase in the respective standard deviation occurred in some cases with the increment of GLCM size. It was found that this occurred when the GLCM sizes were 32×32 and 64×64 for the 16 GLCM/neuron group and when the GLCM size was 64×64 for the 32 GLCM/neuron group. The only time the 64×64 GLCM size produced a good result was for the 60 GLCMs/neuron group. Due to this, the sensitivity values could not be calculated for those cases. Therefore, the AUC values also dropped from 99.96% to 89.3% in the GM–GLCM image training method. It was found that the GD–GLCM image training method maintained a consistently high AUC value of 99.96% for all cases, in comparison to the standard method, which could only provide ~86% AUC values. Thus, for the 1-hidden layer MLP the GD–GLCM image training method provided the best performance.

It was found that for a two hidden layer MLP, that accuracy was in the range of (69.04% ±3.37% to 72.1% ± 7.3%) were produced for the standard method; (95.23% ±2.8% to 99.87% ± 0.81%) for the GM–GLCM method and (98.03% ±1.5% to 99.96% ± 0.09%) for the GD–GLCM method. GM–GLCM and GD–GLCM consistently have 99.96% AUC values, while the standard method has ~86% AUC values. Thus, it was found that there was no significant drop in sensitivity, selectivity, accuracy or values when a two-hidden layer MLP was used as opposed to a 1-hidden layer MLP.

Regarding GLCM size, we *hypothesised* that with a gradual increment of GLCM size (i.e., 8×8 to 16×16 to 32×32 to 64×64), at one point, the pattern will be diluted enough for our classifier to fail. It will occur earlier when we are generating less information (i.e. creating fewer GLCMs) from each neuron. The observation that the sudden drop occurs at 32×32 and 64×64 GLCM sizes for 16 GLCM/neuron group, at a 64×64 GLCM size for the 32 GLCM/neuron group and not at all for the 60 GLCM/neuron group proved our *hypothesis*. We did not, however, observe any sudden drop for the GD–GLCM image training method, though. This was because the spatial arrangements of the patterns that existed between healthy and dying cells were much more pronounced using GD than GM (as shown in Fig 4). Therefore, the GM–GLCM image training method requires a ’two hidden layers MLP’ for increasing GLCM size, whereas the GD–GLCM image training method serves to reduce the overall complexity of the problem requiring only a ’one hidden layer MLP’ to solve.

Thus, in conclusion, the GD–GLCM image training method achieved the highest performance of 99.96% AUC over the other 2 methods presented. In addition, the GD–GLCM image training method also provided: the most minimised MLP architecture–only requiring a 1-hidden-layer MLP, the most minimised input space–only requiring an 8×8 GLCM sizes for input and the most minimised training data of 16 GLCMs/cell group. This new automated GD-GLCM image training method is significant as it can now be used to classify healthy and dying cortical cells in hypoxic-ischemic fetal sheep brain slice images rapidly, speeding up the histological analysis process over the manual assessment that is currently used and only requires small training data and shallow learning to be able to do so.

## Supporting information

S1 File Tabulation data for Fig 7. (XLSX)

S2 File Tabulation data for Fig 8. (XLSX)

S3 File Tabulation data for Fig 9. (XLSX)

S4 File Tabulation data for Fig 10. (XLSX)

S5 File Tabulation data for Fig 11. (XLSX)

S6 File Tabulation data for Fig 12. (XLSX)

S7 File Tabulation data for Fig 13. (XLSX)

S8 File Tabulation data for Fig 14. (XLSX)

S9 File Tabulation data for Fig 15. (XLSX)

S10 File Tabulation data for Fig 16. (XLSX)
